# Transthyretin as a Biomarker to Predict and Monitor Major Depressive Disorder Identified by Whole-Genome Transcriptomic Analysis in Mouse Models

**DOI:** 10.3390/biomedicines9091124

**Published:** 2021-08-31

**Authors:** Sung-Liang Yu, Selina Shih-Ting Chu, Min-Hui Chien, Po-Hsiu Kuo, Pan-Chyr Yang, Kang-Yi Su

**Affiliations:** 1 Department of Clinical Laboratory Sciences and Medical Biotechnology, College of Medicine, National Taiwan University, Taipei 10051, Taiwan; slyu@ntu.edu.tw (S.-L.Y.); r98424014@ntu.edu.tw (S.S.-T.C.); julia861009@hotmail.com (M.-H.C.); 2 Department of Laboratory Medicine, National Taiwan University Hospital, Taipei 10051, Taiwan; 3 Graduate Institute of Pathology, College of Medicine, National Taiwan University, Taipei 10051, Taiwan; 4 Center for Optoelectronic Biomedicine, College of Medicine, National Taiwan University, Taipei 10051, Taiwan; 5 Department of Public Health, Institute of Epidemiology and Preventive Medicine, College of Public Health, National Taiwan University, Taipei 100, Taiwan; phkuo@ntu.edu.tw; 6 Department of Internal Medicine, College of Medicine, National Taiwan University, Taipei 10051, Taiwan; pcyang@ntu.edu.tw; 7 Genome and Systems Biology Degree Program, College of Life Science, National Taiwan University, Taipei 10617, Taiwan

**Keywords:** major depressive disorder, transthyretin, chronic mild stress, transcriptome, amygdala

## Abstract

Background: Accumulations of stressful life events result in the onset of major depressive disorder (MDD). Comprehensive genomic analysis is required to elucidate pathophysiological changes and identify applicable biomarkers. Methods: Transcriptomic analysis was performed on different brain parts of a chronic mild stress (CMS)-induced MDD mouse model followed by systemic analysis. QPCR and ELISA were utilized for validation in mice and patients. Results: The highest numbers of genes with significant changes induced by CMS were 505 in the amygdala followed by 272 in the hippocampus (twofold changes; FDR, *p* < 0.05). Enrichment analysis indicated that the core-enriched genes in CMS-treated mice were positively enriched for IFN-γ response genes in the amygdala, and hedgehog signaling in the hippocampus. Transthyretin (TTR) was severely reduced in CMS-treated mice. In patients with diagnosed MDD, serum concentrations of TTR were reduced by 48.7% compared to controls (*p* = 0.0102). Paired samples from patients with MDD demonstrated a further 66.3% increase in TTR at remission compared to the acute phase (*p* = 0.0339). Conclusions: This study provides comprehensive information on molecular networks related to MDD as a basis for further investigation and identifies TTR for MDD monitoring and management. A clinical trial with bigger patient cohort should be conducted to validate this translational study.

## 1. Introduction

Major depressive disorder (MDD) is a potentially life-threatening disorder with a complicated etiology that imposes a major public health and economic problem worldwide. According to a World Health Organization (WHO) report, it may become a leading cause of disability by the end of 2030 [1]. Patients with MDD exhibit a diverse range of symptoms, including psychomotor retardation, agitation, reduced motivation, depressed mood, and suicidality [2,3]. An epidemiological survey conducted on the working population highlighted the correlations between both chronic psychosocial stress and depression with heart disease, high blood pressure, and high blood glucose [4]. Stress and depression both cause neural atrophy and apoptosis in different areas of the brain, including reduction in the numbers and size of neurons and glial cells, and decreased neurogenesis in the prefrontal cortex (PFC) or hippocampus of patients with depressive disorders [5]. Stress can also affect brain function and inflammatory activity by enhancing the expression of cytokines or suppressing the immune system [6,7,8]. In addition, it can increase activation of the hypothalamus–pituitary axis (HPA) and promote depressive-like behavior [9]. Therefore, several components of the HPA axis are involved in the development of MDD-related changes in the hippocampus, amygdala, and PFC [10]. However, the detailed molecular mechanism underlying these changes, as well as the pathogenesis of MDD, including onset and progression, have not yet been fully clarified due to the wide spectrum of symptoms and complicated etiology.

The prediction, diagnosis, and treatment of MDD has become an emerging issue, where current antidepressants are only effective in approximately 40–50% of patients [11]. In addition to subjective criteria, objective biomarkers that reveal the pathophysiological changes specific to MDD are required to ensure that effective treatments are developed. Therefore, an animal model of MDD may facilitate the development of applicable biomarkers, novel drug discovery, and pre-clinical trials. Until now, the validated mouse model of human MDD induced by the unpredictable chronic mild stress (CMS) had been widely utilized. However, it is also subject to variability related to animal strain, environmental factors, the strength and frequency of treatment, stressor variety, and technical diversity, all of which are still unable to be avoided [12]. Several previous reports have highlighted that CMS reduces aggressiveness and male sexual behavior, and generates both behavioral and physiological abnormalities characteristic of human depression. One previous study suggested that CMS is associated with behavioral changes, body weight loss, and learning and memory impairments [13]. Another study using microarray analysis showed that CMS affects several genes in the cerebral cortex and hippocampus, and that these genes are involved in multiple functions, such as the regulation of neurotransmitters and growth factors [14]. Further to this, using microarray analysis, Yamanishi et al. reported that Hnf4a directly affects various genes associated with several metabolic processes in the prefrontal cortex [15]. Therefore, a comprehensive, systemic, and comparative analysis of different brain areas involved in emotion and the stress response should be conducted. In addition, developing a potential biomarker to predict MDD and monitor outcomes from genomic studies would be extremely beneficial; however, this remains to be identified. In this study, we provided comprehensive genomic information related to MDD, and identified transthyretin (TTR) as a gene significantly downregulated in most brain areas as well as in the peripheral blood of CMS-treated mice. Importantly, we found TTR to be significantly elevated in the serum of MDD patients during the remission phase compared to paired serum samples from the acute phase. Thus, this study identified TTR as a possible biomarker for MDD monitoring.

## 2. Materials and Methods

### 2.1. Animals

Six- to eight-week-old adult male C57BL/6 mice were purchased from the National Taiwan University Laboratory Animal Center. To minimize variation, the mice were allowed to remain in the transport cage for seven days prior to stress induction. Mice had free access to food and water, and were housed at 22 ± 1 °C with 50 ± 2% humidity and a 12 h light/12 h dark schedule, except for when the CMS procedure required continuous overnight illumination. All experiments were carried out with the agreement and approval of the Institutional Animal Care and Use Committee (IACUC) of National Taiwan University Medical College (approval number 20120060).

### 2.2. Chronic Mild Stress Procedures

Mice were randomly divided into two groups (*n* = 6 each), the CMS group and the control group. The CMS experimental group underwent unpredicted unique or combined stressors exposure 6 days a week for 4 weeks. All CMS treated mice experienced the same sequence of stressors. The stressors consisted of the followings.

#### 2.2.1. Fear Stress

Mice were housed together with a rat. They were introduced in a customized metallic mesh cage (21 cm × 18 cm × 14 cm), which were place in the central of a rat home space (60 cm × 45 cm × 30 cm). The exposed time was about 8 to 12 h; food and water were freely available.

#### 2.2.2. Cage Tilting

Mice were placed in a 45° tilted cage for 2 or 8 h according to CMS schedule.

#### 2.2.3. Cage Shaking

Mice were placed in the cage on the shaking machine with 80 to 100 rpm horizontal shaking for 10 min and rest for next 10 min. The procedure was repeated 6 times within 2 h period.

#### 2.2.4. Wet Sawdust Bedding

Three hundred ml water was added to wet the bedding and mice were placed in this cage for 2 or 8 h according to the CMS schedule.

#### 2.2.5. Physical Restraint and Social Stress

Mice were separated individually in a restraint space for physical restraint stress and ten mice were placed together in one cage for social stress. The duration was according to the CMS schedule. During treatment, mice had free access to food and water.

#### 2.2.6. Water Emergency

The mice were placed in another cage without sawdust bedding and filled with tepid water (about 2 cm in depth) for 10 min and rest for next 10 min. The procedure was repeated 6 times within 2 h period.

#### 2.2.7. Tail Pinching

A 3 cm long artery clip with the branches of 1 cm long, 1.5 mm in diameter is used to press the base of mice’s tail for ten minutes. This was repeated 6 times at 10 min intervals.

#### 2.2.8. Continuous Overnight Illumination

Mice mere housed in a separated cage with converted dark to light cycle for 12 h.

### 2.3. Behavioral Testing Assessments

#### 2.3.1. Running Wheel Test

Mice were placed into a customized running wheel (5 cm wide, 15 cm diameter) and allowed to run freely for five minutes. Running cycles were counted manually.

#### 2.3.2. Forced Swimming Test (FST)

The FST was performed according to the protocol of a previous study [16]. Briefly, mice were placed in a glass cylinder (45 cm high, 15 cm diameter) filled with water to a depth of 30 cm (23–25 °C). Water was changed between each mouse test. Immobility time was recorded over a 6 min period with a digital camera and analyzed using DepressionScan Suite software (CLEVER Sys. Inc., Reston, VA, USA).

#### 2.3.3. Tail Suspension Test (TST)

The TST was carried out according to the protocol of a previous study [17]. Briefly, mice were securely fastened by the 1.0–1.5 cm tip of their tail to a flat metallic surface using medical adhesive tape and suspended ~30 cm above the ground in a 40 cm^3^ box, thus isolating the mice from visual distractions while permitting observation of their behavior from above. Immobility time was defined as the absence of limb or body movement within the 6 min testing period. Criteria were defined and data analyses were performed using DepressionScan Suite.

### 2.4. Expression Microarray Experiments and Data Analysis

The GeneChip Mouse Genome 430 2.0 Array (Affymetrix, Santa Clare, CA, USA) was used to perform whole genome expression profiling. The procedures were performed according to the manufacturer’s manual. Briefly, 100 ng of pooled RNA was used for the Two-Cycle cDNA Synthesis Kit. Biotin-labelled cRNA was produced through in vitro cDNA transcription (IVT Labeling Kit, Affymetrix). Fragmented cRNA (20 μg) was hybridized to the chip by using the Affymetrix Fluidics Station 450 (Affymetrix). The arrays were washed and stained according to the supplied protocols followed by GeneChip Scanner 3000 (Affymetrix) scanning. The raw data contained in the CEL file were further analyzed using GeneSpring GX software (Agilent Technologies, Santa Clare, CA, USA) with the GCRMA summarization algorithm and baseline to mean normalization. The original CEL files, normalized data, and experimental information were deposited in the Gene Expression Omnibus (accession number GSE151807). Significant genes were selected by statistical analysis performed with false discovery rate (FDR) correlated *p* value < 0.05 and fold change greater than 2 in CMS treated mice compared with control mice for Metacore software (Thomson Reuters) pathway analysis. The gene expression data generated by cDNA expression microarray were analyzed using GSEA 4.1.0 (http://software.broadinstitute.org/gsea/index.jsp, accessed on 30 July 2020) to extract biological knowledge. Highly significant enriched gene sets are shown. The false discovery rate (FDR) is calculated by comparing the actual data with 1000 Monte Carlo simulations. The normalized enrichment score (NES) computes the density of modified genes in the data set with the random expectancies, normalized by the number of genes found in a given gene cluster, to consider the size of the cluster.

### 2.5. QPCR Validation

Quantitative PCR (QPCR) was performed in a 96-well format. The SYBR green primers of the genes were designed by Primer Express 3.0. The cDNA amplification was carried out using an ABI 7700 Sequence Detection System (Applied Biosystems), and the detection was carried out by measuring the binding of the fluorescence dye SYBR Green I to double-stranded DNA. The relative expression level of the target gene compared with that of the mouse TBP (TATA box binding protein) was defined as −ΔCT = (CTgene − CTmTBP). The gene mRNA/mTBP mRNA ratio was calculated as 2^−ΔCT^. The primers used for qPCR are listed in Supplementary Table S1.

### 2.6. Immunohistochemical (IHC) Staining

Mice brains at the end of experiments were isolated for post fixation overnight in the 4% paraformaldehyde at 4 °C and embedded in paraffin. Five-micrometer-thick coronal sections were deparaffinized followed by epitope retrieval with citrate buffer. After treating with 3% H_2_O_2_, primary antibodies were incubated for overnight at 4 °C. Immunostaining was performed by liquid DAB-substrate-chromogen system (DAKO,Cat. K3467, Santa Clare, CA, USA) for 10 min and counterstain with hematoxylin (Sigma-Aldrich, Cat. GHS316, Burlington, MA, USA) for 10 s. Slides were stained with anti-Tau1 (Sigma-Aldrich, Cat. MAB3420, Burlington, MA, USA), anti-MAP2 (Abcam, Cat. ab5392), anti-synapsin (Abcam, Cat. ab64581, Cambridge, UK), and anti-ki-67 (Abcam, Cat. ab15580, Cambridge, UK) antibodies.

### 2.7. TTR ELISA

TTR ELISA was performed by Enzyme-linked Immunosorbent Assay Kit for Transthyretin (TTR) (Cat. E90726Mu) (Uscn Life Science, Wuhan, China) according to the user manual.

### 2.8. Patient Samples

Twelve patients who were diagnosed with MDD according to the criteria of the Diagnostic and Statistical Manual of Mental Disorders, 5th version (DSM-5) and had sufficient specimens for testing were consecutively referred to the study by psychiatrists from central and regional hospitals in Taipei. Twelve case-control participants were obtained from the community in matched catchment areas. Case-controls had no past diagnosis of major psychiatric disorders, including anxiety disorder, mood disorder, schizophrenia, mental retardation, or substance use disorder. Acute manic patients were followed until they achieved full remission or for at least 2 months. In total, six patients with paired and sufficient serum samples were collected for testing. All participants signed informed consent forms after the study procedures were fully explained. The tests used in this study were approved by the institutional review board of all participating hospitals.

### 2.9. Statistical Analysis

All results are shown as the mean ± standard deviation (SD) of *n* observations. The sample size was determined based on the results from pilot studies and previous experience regarding the variability of each data set within experimental and control groups. Each experiment was repeated at least three times, resulting in the same conclusion. Data analysis was performed using GraphPad Prism 8. Statistical analysis for behavioral tests was performed using two-way ANOVA and mixed-effects model. Statistical analysis for expression data was performed using an unpaired, two-tailed Student’s *t-*test. TTR concentrations in paired patient serum samples were analyzed using a paired, two-tailed Student’s *t*-test. Differences between the means of two compared groups considered to be statistically significant were denoted as * *p* < 0.05, ** *p* < 0.01, and *** *p* < 0.001.

## 3. Results

### 3.1. Mice That Underwent CMS Gained Less Body Weight and Exhibited Reduced Mobility

**Figure 1:**
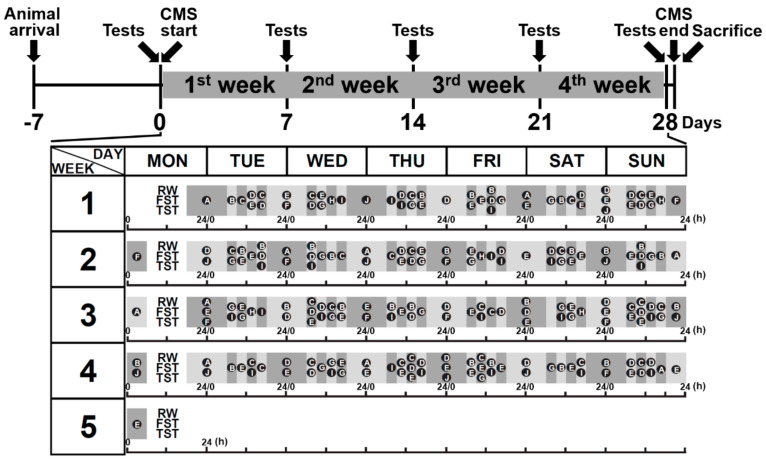
The induction schedule of chronic mild stress (CMS) treatment. Mice were allowed to remain in cages for a week before stress induction to eliminate the environment as a potential confounding factor. According to the protocol, CMS-treated mice underwent unpredicted unique or combined stressor exposure at a frequency of 6 days per week for 4 weeks. All CMS-treated mice experienced the same sequence of stressors; meanwhile, control mice were housed in standard conditions. At the end of the experiments, mice were sacrificed, and brain areas including the amygdala, hippocampus, prefrontal cortex, and cerebral cortex were isolated and collected. A, housing of rats; B, 45° cage tilt; C, cage shaking every 10 min; D, wet sawdust bedding; E, physical restraint; F, flash lighting; G, water emergency every 10 min; H, physical and social stress; I, tail pinching every 10 min; J, continuous overnight illumination; RW, running wheel; FST, forced swimming test; TST, tail suspension test.

**Figure 2:**
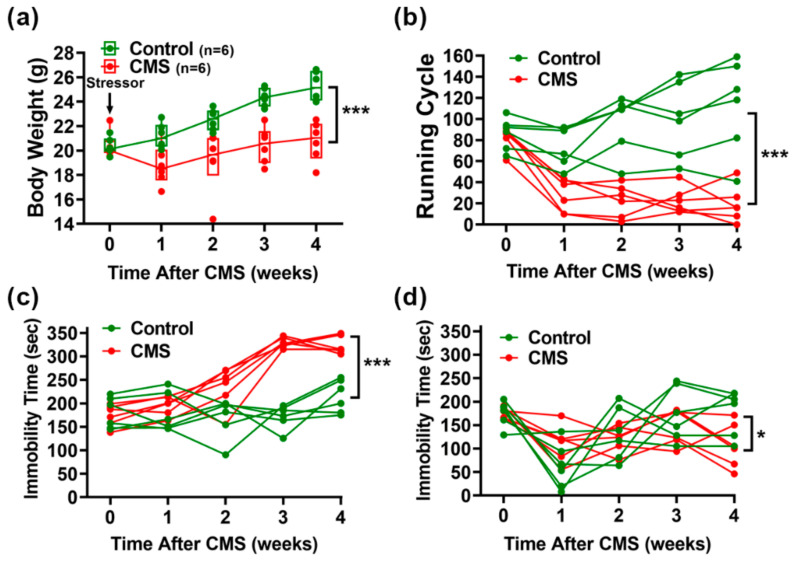
Behavioral tests of mice after CMS stimulation. (**a**) Body weight recording during 4-week CMS treatments. (**b)** Running wheel test results. Mice were placed on the running wheel for 5 min. Running cycles were recorded using a video camera then counted manually. (**c**) Forced swimming test results. Mice were placed in a glass cylinder filled with water for 6 min. Their time spent immobile was recorded, followed by analysis with DepressionScan Suite software. (**d**) Tail suspension test results. Mice were placed in a box to isolate them from visual distractions, and securely fastened and suspended for 6 min. Their time spent immobile was recorded, followed by analysis with DepressionScan Suite software. Statistical analyses were performed using two-way ANOVA and mixed-effects model (*n* = 6 for CMS and control groups). The significant interaction between time and CMS treatment was analyzed. *, *p* < 0,05; ***, *p* < 0.001.

The CMS procedure involved the combination of ten various, unpredictable, mild stressors (Figure 1). To evaluate the induction efficacy of CMS, body weight monitoring and behavioral tests, including the running wheel test (RWT), forced swimming test (FST), and tail suspension test (TST), were performed before and each week after CMS treatments according to the schedule (Figure 2). Mice that underwent CMS (*n* = 6) gained significantly less weight over the 4-week treatment period compared to controls (*n* = 6; Figure 2a). Using ANOVA, time [F (4, 40) = 23.00, *p* < 0.001] and CMS treatment [F (1, 10) = 20.42, *p* < 0.01] were identified as significant main effects. In addition, a significant interaction was identified between time and CMS treatment [F (4, 40) = 10.31, *p* < 0.001]. CMS was also found to significantly reduce motor activities according to a RWT performed in CMS mice (*n* = 6) compared to control mice (*n* = 6; Figure 2b). Again, ANOVA revealed time [F(4, 40) = 7.86, *p* < 0.001] and CMS treatment [F (1, 10) = 27.88, *p* < 0.001] to be significant main effects. In addition, a significant interaction was found between time and CMS treatment [F (4, 40) = 15.88, *p* < 0.001]. Significant increases in immobility during the FST and TST were also observed in CMS mice (*n* = 6) compared to control mice (*n* = 6; Figure 2c,d). ANOVA revealed time [F (4, 40) = 20.82, *p* < 0.001] and CMS treatment [F (1, 10) = 61.47, *p* < 0.001] to be significant main effects, and an interaction between time and CMS treatment [F (4, 40) = 14.05, *p* < 0.001] in FST was also found. Meanwhile, a significant main effect of time [F (4, 40) = 7.739, *p* < 0.001] and an interaction between time and CMS treatment [F (4, 40) = 3.164, *p* = 0.0238] were observed for TST, but no significant effect was found for CMS treatment [F (1, 10) = 1.209, *p* = 0.2972].

### 3.2. Histopathological Analysis of CMS-Treated Mouse Brains

To test the impact of CMS on histopathological and anatomical brain changes, immunohistochemical staining was performed on axons and dendrites using antibodies against Tau-1 and MAP2, respectively (Supplementary Figures S1 and S2). No obvious significant abnormality in neurite structures was found. However, the MAP-2-positive neurites in the hippocampus CA1 region of CMS-treated mice seemed to be mildly shrunken and disorganized in appearance. There were no obvious differences between CMS and control mice.

### 3.3. Identification of MDD-Related Gene Signatures Using Whole-Genome Transcriptomic Analysis

**Figure 3:**
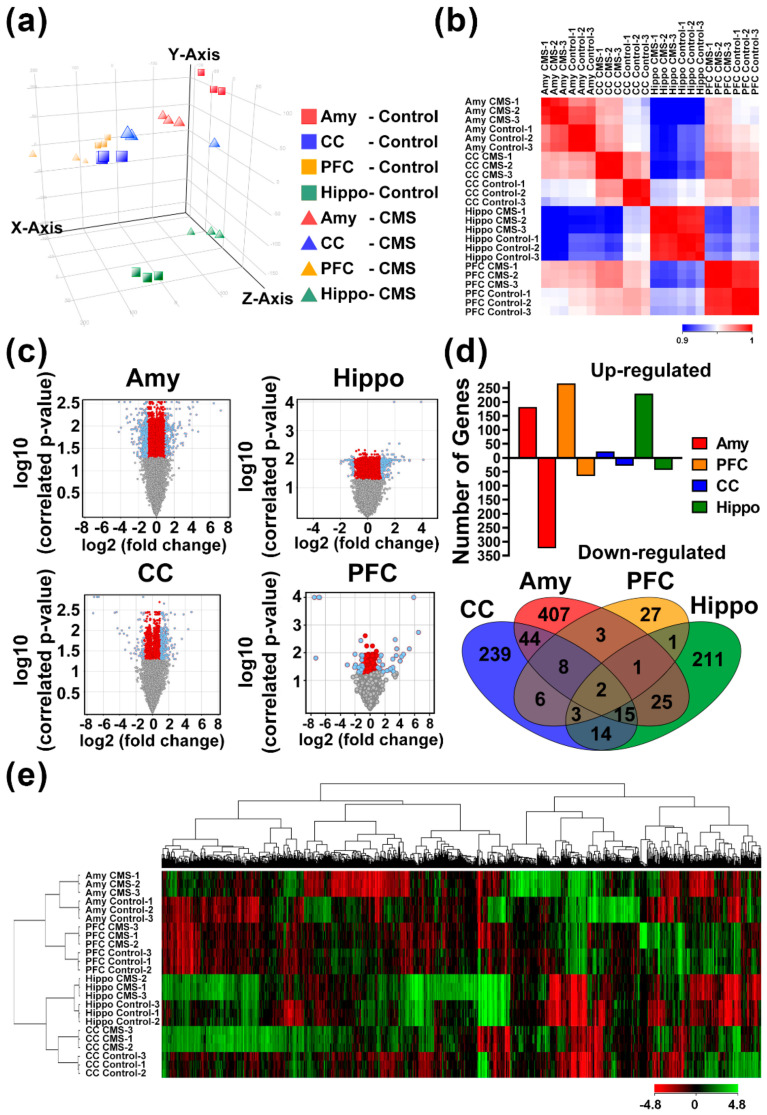
Whole-genome transcriptomic analysis for genes with significant alterations in brain areas due to chronic mild stress (CMS) stimuli. Brain areas from CMS and control mice (*n* = 3 for each group, biological repeats) were assessed for expression microarray experiments followed by Genespring (Agilent Technologies, Santa Clara, CA, USA) software analysis. (**a**) PCA correlation of individual samples. (**b**) Pearson correlation coefficient-based heat map of individual experiments. (**c**) Volcano plot of log2 fold change versus log10 false discovery rate (FDR)-correlated *p* value for all genes among four brain areas. Blue dots represent significant correlated genes with FDR *p* < 0.05 and fold-change >2. (**d**) Comparison of genes with significant changes and Venn diagram illustration of the four brain areas. (**e**) Heat map diagram with two-way unsupervised hierarchical clustering of significant union genes and samples. Each column represents a gene and each row represents a sample. The gene clustering tree is shown on the top, and the sample clustering tree is shown on the left. The color scale shown in the map illustrates the relative expression level of a gene across all samples: red represents an expression level above the mean and green represents expression lower than the mean. Amy, amygdala; hippo, hippocampus; PFC, prefrontal cortex; CC, cerebral cortex.

Whole-genome transcriptomic analysis was performed using expression cDNA microarrays to identify genetic biomarkers associated with MDD-like behavior in the CMS-induced mouse model. Brain areas, including the amygdala (Amy), hippocampus (Hippo), prefrontal cortex (PFC), and cerebral cortex (CC), were separated into independent analyses comparing CMS and control mice. Each brain area from three independent mice in both the CMS and control groups was assessed in the described assays, and data were analyzed (Figure 3). Principal component analysis (PCA) was used to illustrate the global gene expression changes among the different areas and treatments (Figure 3a). A Pearson correlation cluster heatmap showed that the gene expression profiles were more similar between CMS treatments than between brain areas (Figure 3b). Interestingly, the hippocampus exhibited relatively unique patterns in comparison with other areas. Volcano plot analysis demonstrated that probes of genes were significantly correlated in each of the four brain areas (FDR *p* < 0.05 and over twofold change [blue dots]; Figure 3c). Among these brain areas, the amygdala exhibited the greatest number of probes with significant changes in abundance. Probes of genes with significant changes included the following: 182 upregulated and 323 downregulated in Amy; 266 upregulated and 65 downregulated in CC; 229 upregulated and 43 downregulated in Hippo; and 23 upregulated and 28 downregulated in PFC (Figure 3d). The expression levels of the top ten CMS-affected genes in all brain areas ranged from 39.30 to 3.94 among upregulated genes and 100.00 to 2.33 among downregulated genes (Supplementary Table S2). A Venn diagram was generated to visualize the intersecting and unique genes that were significantly affected among the four brain areas (Figure 3d). Furthermore, 16 genes with significant changes were present in at least three brain areas, where S100a8 commonly affected by CMS in all four brain areas. A heat map was created with unsupervised clustering using a total of 1006 probes with significant changes in the four brain areas, and demonstrated that all conditions were well differentiated according to treatment groups and brain areas (Figure 3e).

### 3.4. GSEA Predicts Potential Biological Function Related to CMS Induction

**Figure 4:**
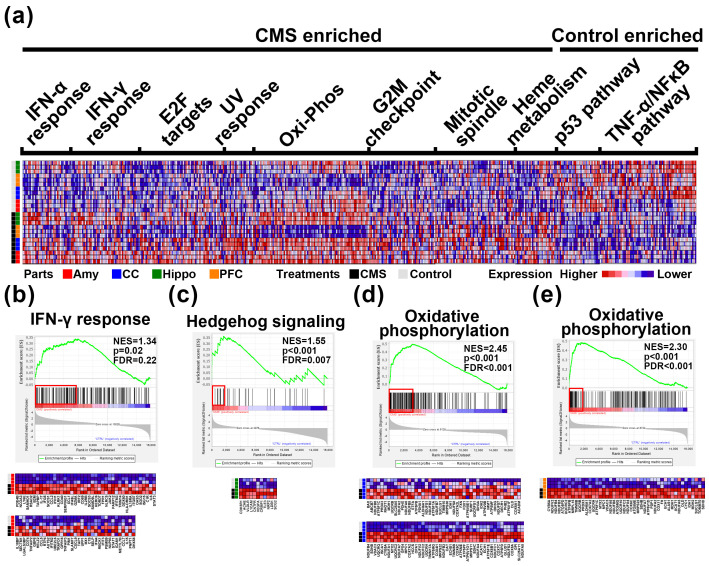
Gene set enrichment analysis (GSEA) of expression results in brain areas induced by chronic mild stress (CMS) stimuli. (**a**) Heat map showing core enriched gene clusters involved in several significant differences in hallmark signatures among the four brain areas, including IFN-α response, IFN-γ response, E2F targets, UV response, oxidative phosphorylation, G2M checkpoint, mitotic spindle, heme metabolism, p53 pathway, and TNF-α signaling. (**b**) Graphical representation of core-enriched genes in the CMS-treated amygdala showed positive enrichment for the IFN-γ response. (**c**) Graphical representation of core-enriched genes in the CMS-treated hippocampus showed positive enrichment for Hedgehog signaling. (**d**) Graphical representation of core-enriched genes in the CMS-treated CC showed positive enrichment for oxidative phosphorylation. (**e**) Graphical representation of core-enriched genes in the CMS-treated PFC showed positive enrichment for oxidative phosphorylation. Amy, amygdala; hippo, hippocampus; PFC, prefrontal cortex; CC, cerebral cortex; NES, normalized enrichment score; FDR, false discovery rate.

To clarify the potential underlying pathways involved in CMS stimulation, GSEA was further utilized as a means to identify hallmark gene signatures differentially regulated by stimuli (Figure 4 and Supplementary Table S3). According to combined four parts analysis, we identified several core gene clusters which were enriched in CMS-treated or control mice and were involved in several hallmarks among the four brain areas (Figure 4a). Among these hallmarks, specifically, the most enriched hallmark in Amy (Figure 4b), CC (Figure 4d), and PFC (Figure 4e) of CMS-treated mice were IFN-γ response, oxidative phosphorylation, and oxidative phosphorylation, respectively. Interestingly, Hippo was a unique part with only a hallmark enriched in hedgehog signaling (Figure 4c).

### 3.5. Pathway Prediction and Regulatory Network Construction in CMS-Treated Mice

**Figure 5:**
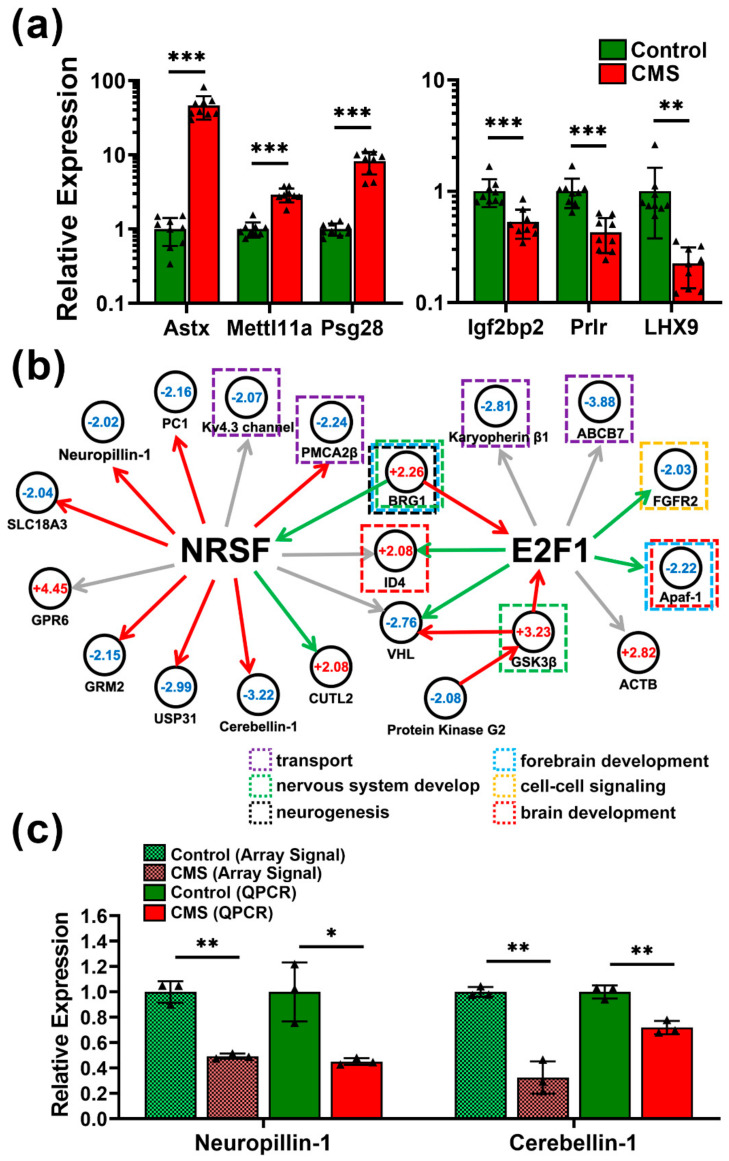
qPCR validation and network construction for underlying chronic mild stress (CMS)-induced molecular regulation. (**a**) qPCR validation for CMS-induced genes with significant alterations. Top three upregulated genes (**left** panel) and top three downregulated genes (**right** panel) in the CMS-treated amygdala. (**b**) Regulatory network construction based on transcriptional regulation. Genes displaying significant changes in the CMS-treated amygdala were subjected to further pathway analysis using Metacore software. NRSF and E2F functioned as two core transcription factors under BRG1 and GSK3β stimulation, respectively. Fold changes in gene expression are indicated with numbers, and genes with functions related to neurodevelopment and neurogenesis are highlighted. Green arrows indicate positive regulation/activation, red arrows indicate negative regulation/inhibition, and gray arrows indicate inconclusive regulation. (**c**) qPCR validation for neurite growth-related genes, neuropillin-1 and cerebellin-1. The results from both the expression array and qPCR were compared in parallel. GPR6, G protein-coupled receptor 6; GRM2, glutamate receptor metabotropic 2; USP31, ubiquitin specific peptidase 31; KCNQ5, potassium voltage-gated channel KQT-like subfamily 5; CUTL2, cut-like homeobox 2; PMCA2b, plasma membrane calcium ATPase 2; SLC18A3, solute carrier family 18; PC1, proprotein convertase subtilisin/kexin type 1; BRG1, BRM/SWI2-related gene 1; ID4, inhibitor of DNA binding 4; VHL, von Hippel-Lindau tumor suppressor; ACTB, beta-actin; Apaf-1, apoptotic peptidase activating factor 1; FGFR2, fibroblast growth factor receptor 2; ABCB7, ATP-binding cassette B7. Statistical analysis was performed using unpaired, two-tailed Student’s *t* tests. * *p* < 0.05, ** *p* < 0.01, and *** *p* < 0.001.

Metacore software was utilized to analyze regulatory signaling affected by CMS in all four brain areas. The differentially expressed genes were then categorized according to process networks, disease, and GO processes (Supplementary Table S4). The top ten significantly enriched terms were listed for each brain area after analysis using a threshold of FDR *p* < 0.01. The results indicated that CMS may affect several developmental processes in neurons and in the immune system. The most abundant gene alterations were found in the amygdala; therefore, we focused on this area for further analysis and regulatory network construction. Firstly, we validated transcriptomic results in Amy using qPCR (Figure 5a). The top three upregulated and downregulated genes in Amy also demonstrated significant changes, consistent with the transcriptomic results. The underlying regulatory network was constructed based on the transcriptional regulation module. We determined that two core transcription factors, NRSF and E2F1, which are downstream of BRM/SWI2-related gene 1 (BRG1) and GSK3β, respectively, had direct interactions with many genes related to neuronal functions (Figure 5b). Specifically, CMS may affect processes including cell–cell signaling, brain development, intracellular transport, neurogenesis, forebrain development, and nervous system development via modulation of NRSF and E2F1. Moreover, two important genes in this network, neuropillin-1 and cerebellin-1, related to neurite outgrowth were significantly downregulated in CMS-treated mice (Figure 5c), which further validates the impact of CMS on neurite trajectory observed through IHC staining (Supplementary Figures S1 and S2).

### 3.6. Transthyretin (TTR) Is a Biomarker for MDD Monitoring

**Figure 6:**
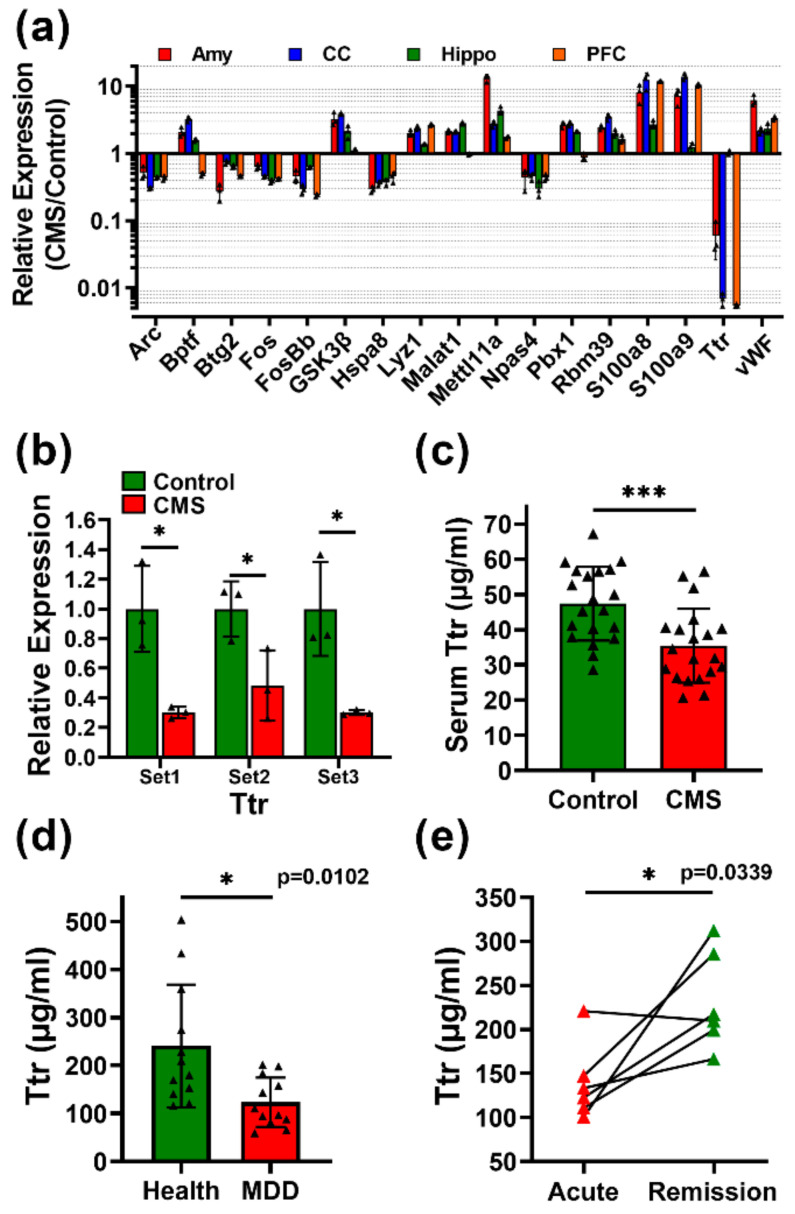
Transthyretin (TTR) is a serum biomarker for major depressive disorder (MDD). (**a**) Expression profiling of genes with significant changes and consistent trends in response to CMS treatment in at least three of the four brain areas. TTR exhibited significant downregulation in the amygdala, prefrontal cortex, and cerebral cortex after CMS treatments. (**b**) qPCR validation of TTR expression in the amygdala using three independent sets of primers. (**c**) Serum TTR concentrations in CMS-treated and control mice according to ELISA (*n* = 20 independent mouse serum samples from three independent CMS treatments per group). (**d**) Serum TTR concentrations in patients with diagnosed MDD and case-controls. (**e**) TTR concentrations in paired serum samples from patients at acute and remission phases of MDD. Statistical analysis was performed using an unpaired, two-tailed Student’s *t*-test (**d**) and a paired, two-tailed Student’s *t*-test (**e**). Amy, amygdala; hippo, hippocampus; PFC, prefrontal cortex; CC, cerebral cortex. * *p* < 0.05, and *** *p* < 0.001.

The identification of an applicable biomarker for MDD is necessary to improve diagnosis and monitoring in clinical settings. We filtered the results of the transcriptomic analysis to identify genes with a greater than twofold change in at least three of the four brain areas based on the previous analysis (Figure 3d). A total of 17 genes were selected for further analysis (Figure 6a). Notably, TTR was dramatically reduced in Amy, CC, and PFC after CMS treatment. We further confirmed this by three independent qPCR primer sets in Amy highly related to emotion regulation (Figure 6b). ELISA also validated the finding of reduced serum TTR concentrations in CMS-treated mice (Figure 6c). Finally, we assessed whether the TTR concentration in the serum could distinguish patients with MDD (Supplementary Table S5; Figure 6d). Patients with diagnosed MDD were found to exhibit significantly reduced serum TTR compared with health controls. Furthermore, paired serum samples from MDD patients in acute and remission stages showed that TTR levels were significantly enhanced in the remission stage compared with the acute stage (Figure 6e).

## 4. Discussion

Although novel antidepressant therapies are currently emerging, challenges remain due to very limited knowledge of the molecular etiology and clinical complexity of MDD. CMS animal models are valuable tools to help improve understanding of the pathological processes underlying MDD. They can be used to identify specific biomarkers for disease monitoring and support the development of novel antidepressant treatment strategies [18]. In the present study, we found that the amygdala of CMS-treated mice displayed the greatest number of altered genes compared with other areas, which suggests that it was highly affected by CMS. It is not surprising that the amygdala exhibited the greatest number of gene expression changes following CMS treatment, since it plays a crucial role in regulation of the stress response and mediates the influence of stress on memory consolidation and recall [19]. Patients with depression and anxiety have been found to exhibit specific amygdala reactivity [20]. Furthermore, the amygdala represents the node of an extended corticolimbic circuit that supports emotion processing and stress responsiveness [21]. It interacts with other areas in the brain, including the hippocampus and prefrontal cortex, to promote enhancement of several forms of perceptual processing as well as attention, which serve to create memory of emotional material [22]. Accumulating evidence has shown that in the hippocampus, chronic stress may alter cellular functions and plasticity of neurons, and result in dendritic atrophy as well as inhibition of neurogenesis [23]. In particular, chronic stress can also induce dendritic atrophy and reduce the length of apical dendrites of pyramidal neurons in the CA1 and CA3 regions, which may reduce neuroplasticity [24,25,26]. Furthermore, since GABAergic interneurons of the hippocampus are important in the regulation of cellular and neural circuit function, their roles in the pathogenesis of MDD have been well documented [27]. Our results indicate that neurite outgrowth may also be affected by CMS.

In CMS-induced physiological and functional alterations, firstly, based on GSEA results, gene changes related to CMS in the amygdala are highly enriched in IFN-γ responses (Figure 4b). This indicates that the immune system and inflammatory response of mice may be induced by CMS. It has been reported that subjects with depression exhibit elevated levels of inflammatory immune activation [28]. Increases in pro-inflammatory cytokines, especially IL-1, IL-6, TNF-α, and IFN-γ, may exert neurotoxic effects on specific brain areas implicated in emotional regulation, including the amygdala, hippocampus, and cerebral cortex [29]. Secondly, sonic hedgehog signaling (Shh) was found to be significantly enriched in the hippocampus (Figure 4c). This is supported by a previous study, which found that impaired Shh signaling contributes the pathogenesis of several neurological disorders, including MDD [30]. Thirdly, both PFC and CC exhibited enrichment in the oxidative phosphorylation family of genes, suggesting that metabolic alterations may reflect the consequence of CMS stimulation. Accumulated evidence has revealed the link between CMS and alteration of metabolism. For example, stressed animals demonstrate decreased food intake and preference, as well as decreased appetite hormone homeostasis and systematic metabolome changes [31,32]. Upon deeper examination, we found significant changes in gene families related to electron transfer in the mitochondria complexes of stressed mice, suggesting that energy consumption may be affected by CMS (Figure 4d,e).

According to enrichment analysis, significant changed genes were highly correlated with pathways and signaling involved in the neural system (Supplementary Table S4). Specifically, we found alteration in NRSF and E2F1 transcriptional networks under BRG1 gene regulation in the amygdala (Figure 5b). In particular, NRSF exerted direct effects on various genes related to neurogenesis, metabolic processes, axonal guidance, and immunological regulation in the amygdala. The genes downstream of NRSF were also significantly altered in CMS-induced mice. Although NRSF is a well-known transcriptional regulator involved in neurogenesis and differentiation, its role in neurological disorders and diseases such as schizophrenia [33], Alzheimer’s disease [34], mood disorders [35], and other physiological functions remains controversial [36]. From a clinical perspective, NRSF may be a specific molecular target able to be suppressed by lithium, a mood-stabilizing drug [37]. Other previous studies have found, using transcriptional analysis, that the GSK3β-E2F1 axis may be involved in neuronal apoptosis and differentiation [38], as well as tumor growth [39], and has several direct effects on its downstream effectors. This implies that targeting of GSK3β-E2F1 or NRSF signaling may be able to reduce the effects of CMS-induced bipolar disorder.

In terms of the regulatory network, our results show that two important genes, GSK3β and BRG1, were significantly upregulated after CMS administration (Figure 5). This upregulation consequently resulted in downstream gene expression changes related to several neurophysiological functions, predominantly through two core transcription factors, E2F1 and NRSF. Although GSK3β and BRG1 have been suggested to have an integral role in neurophysiological function, the molecular basis remains difficult to characterize in MDD [40,41]. This may support the development of therapeutic targets by identifying potential drugs that can reverse or regulate genes involved in MDD pathogenesis.

It has been demonstrated that stress can induce not only depressive-like behaviors, but also alterations in oxidative stress, inflammation, neurogenesis, DNA damage, and apoptosis, where the reduction of these changes can efficiently reverse psychopathological and behavioral abnormalities [42,43]. Our enrichment analysis results are also consistent with these studies, and indicate that greater numbers of physiological processes are enriched after CMS treatment (Supplementary Table S2). For example, accumulating evidence has shown that MDD is accompanied by the activation of immune/inflammatory-related cytokines and acute-phase proteins [44,45]. Two meta-analyses have demonstrated a correlation between the expression of several inflammatory cytokines and clinical bipolar disorder using a case-control study [46,47].

This study identified TTR as an applicable biomarker not only for MDD evaluation, but also for disease monitoring (Figure 6). TTR is a protein synthesized and secreted into peripheral blood and CSF, responsible for retinol and thyroxin transportation, and known as prealbumin [13]. It had been reported to associate with several diseases as well as neurological disease. It may aggregate into fibrils and be deposited into the nervous system and result in neuropathy called transthyretin amyloidosis. Some foster the dissociation of TTR tetramer and some facilitate misfolding and denaturation of monomers to cause the irreversible formation of amyloid fibrils. Furthermore, in addition to deposited amyloid fibrils, non-fibrillar circulating formations confer to neurotoxicity and organ disfunction [48]. This can support that lower TTR in peripheral blood may indicate neuropathogenesis. On the other hand, increasing of serum TTR levels by administrating TTR stabilizers may indicate a positive prognostic response to therapy [49]. Patients treated with TTR stabilizers by binding TTR to reduce dissociation of TTR showed reduced morbidity and mortality in a large clinical trial [50]. On the other hand, lower levels of TTR were observed during malnutrition or inflammation as a negative acute-phase reactant [51,52]. This evidence supports our transcriptomic findings that CMS may cause alterations in immune responses, inflammation, and metabolism (Figure 4; Supplementary Table S4) that may be correlated with TTR reduction. Although low TTR may be highly correlated with MDD, and suicidal ideation and low serotonin function in patients have been documented [53,54], the mechanism involved in CMS induced TTR reduction is still enigmatic. It is possible that CMS-induced GSK3β (Figure 5) reduces TTR expression via repressing heat shock factor 1 (HSF1) activation [55,56,57]. Manipulation including exogenous overexpression or endogenous knockdown as well as inhibition by drugs of GSK3β followed by TTR characterization should be considered to reveal the relationship between TTR and GSK3β pathways in the future. This may provide a potential actionable strategy for MDD treatments.

The current study was limited by several factors. Firstly, choosing the most ideal MDD animal model remains a controversial question. Therefore, it cannot be excluded that several confounding factors, such as environments, stressors, animal strains, and treatment strategies, may influence gene expression patterns. We used a CMS strategy with various unpredictable stressors to model MDD. It should be noted that MDD is not a homogenous disorder; it is diagnosed when a certain minimal number of symptoms are displayed in humans. Thus, a limitation of this study was that it may not have allowed clear differentiation of MDD depression subtypes, such as melancholic or atypical depression. Secondly, it should be noticed that MDD is a sex-biased (high occurrence in females) disease. Although only male mice were included for the CMS-induced MDD model, based on the consideration of estrogen interference, it cannot be excluded that some biases may exist. However, the validation in patients (75% females), which indicated that TTR was still highly correlated to MDD, suggested that it may be independent from sex. Thirdly, the sample size of patients with MDD was few and several confounding factors that may affect the power of TTR were difficult to analyze. In order to validate the clinical utilities of TTR, it is necessary to conduct a clinical trial with a large and comprehensive cohort in the future.

## 5. Conclusions

The current study utilized an MDD animal model to comprehensively dissect the underlying molecular mechanisms of MDD. CMS may induce MDD-like pathogenesis accompanied by physiological changes such as IFN-γ response, hedgehog signaling, and oxidative phosphorylation in different brain parts. This provided rationales for further MDD-related investigations. Furthermore, current results also identified TTR as a potential diagnostic and monitoring marker for clinical applications in the future.

## Data Availability

All data generated or analyzed during this study are included in this published article.

## Supplementary Materials

The following are available online at https://www.mdpi.com/article/10.3390/biomedicines9091124/s1. Figure S1: Immunohistochemical staining of Tau-1. Figure S2: Immunohistochemical staining of MAP2. Table S1: Primer List for SYBR Green Quantitative PCR. Table S2: Genes differentially expression in prefrontal cortex, cerebral cortex, amygdala and hippocampus with CMS. Table S3: Gene Enrichment Analysis for CMS vs. Control Mouse Model in Amygdala, Hippocampus, Cerebral Cortex and Prefrontal Cortex. Table S4: Characteristics of patients with MDD and control individuals in the TTR testing cohort.
