# Supplementary material for: Transthyretin as a Biomarker to Predict and Monitor Major Depressive Disorder Identified by Whole-Genome Transcriptomic Analysis in Mouse Models
Source: Biomedicines. 2021 Aug 31;9(9):1124. doi: 10.3390/biomedicines9091124 (PMC8469805; doi:10.3390/biomedicines9091124)
Supplement: Supplementary file 1 [file biomedicines-09-01124-s001.zip › biomedicines-1357577-supplementary.pdf]

Supplementary Material

Transthyretin as a biomarker to predict and monitor major depressive disorder identified by whole-genome transcriptomic analysis in mouse models

Sung-Liang Yu[1,2,3,4], Selina Shih-Ting Chu[1], Min-Hui Chien[1], Po-Hsiu Kuo[5], Pan-Chyr Yang[6] and Kang-Yi Su[1,2,7*]

[1] Department of Clinical Laboratory Sciences and Medical Biotechnology, College of Medicine, National Taiwan University, Taipei, Taiwan, R.O.C; slyu@ntu.edu.tw (S.L.Y); r98424014@ntu.edu.tw (S.T.C); julia861009@hotmail.com (M.H.C.); suky@ntu.edu.tw (K.Y.S)
[2] Department of Laboratory Medicine and, National Taiwan University Hospital, Taipei, Taipei, R.O.C; slyu@ntu.edu.tw (S.L.Y)
[3] Graduate Institute of Pathology, College of Medicine, National Taiwan University, Taipei, Taiwan, R.O.C; slyu@ntu.edu.tw (S.L.Y)
[4] Center for Optoelectronic Biomedicine, College of Medicine, National Taiwan University, Taipei, Taiwan, R.O.C; slyu@ntu.edu.tw (S.L.Y)
[5] Department of Public Health & Institute of Epidemiology and Preventive Medicine, College of Public Health, National Taiwan University, Taipei, Taiwan, R.O.C; phkuo@ntu.edu.tw (P.H.K)
[6] Department of Internal Medicine, College of Medicine, National Taiwan University, Taipei, Taiwan, R.O.C; pcyang@ntu.edu.tw (P.C.Y)
[7] Genome and Systems Biology Degree Program, College of Life Science, National Taiwan University, Taipei, Taiwan, R.O.C; suky@ntu.edu.tw (K.Y.S)
* Correspondence: suky@ntu.edu.tw; Tel.: +886223123456 ext 66910

Supplemental Tables:

Supplemental Table S1: Primer List for SYBR Green Quantitative PCR

Gene Symbol	Description	RefSeq Number	Forward Primer	Reverse Primer
Npr1	Neuropilin-1	NM_008727	5'-CAATCAGAGTTCCCGACATACG-3'	5'-CTGAATCGGCCCTGTCTTG-3'
Cbln1	Cerebellin-1	NM_019626	5'-ATGAGCCGTCCGAGATGAG-3'	5'-TGCGTTCTGAGTCAAAGTTGTTC-3'
Astx	Amplified spermatogenic transcripts X encoded 5	NR_152851.1	5'-CTGATGAAGGAACCCAGGAGAT-3'	5'-AAAAACCTGGCTGACAGTTCCA-3'
Mettl11a	N-terminal Xaa-Pro-Lys N-methyltransferase-1	NM_001356433.1	5'-ACATCTCCAACATCGACCTCAACA-3'	5'-TACCAGCGCCACAGTCCAA-3'
Psg28	pregnancy-specific glycoprotein 28	NM_054063.4	5'-CGTCCAAGTCAACATCCACAAG-3'	5'-AACTGTGCTGTCCGTGACTCTCA-3'
Irf2bp2	Interferon regulatory factor 2 binding protein 2	NM_001164598.2	5'-GGCAGGTTGTTGGGTTTCG-3'	5'-TTTTCCTTGCTGTCCTTGCA-3'
Prlr	Prolacin receptor	NM_011169.5	5'-ATCCCTGGTATGGCAGACTTTTC-3'	5'-CGATGCTCACCTCCACAGAGA-3'
Lhx9	LIM homeobox protein 9	NM_001025565.2	5'-GCAAGGAGGATTATTACAGAAGGTT-3'	5'-CGCGCATGACCATCTCAGA-3'
Ttr	Transthyretin	NM_013697.5	5'-AGCCCTTTGCCTCTGGGAAGAC-3'	5'-TGCGATGGTGTAGTGGCGATGG-3'
Ttr	Transthyretin	NM_013697.5	5'-GACAGGATGGCTTCCCTTCG-3'	5'-CCAGGACTTTGACCATCAGAGG-3'
Ttr	Transthyretin	NM_013697.5	5'-CGTACTGGAAGACACTTGGCAT-3'	5'-GCCGTGGTGCTGTAGGAGTAT-3'
TBP	TATA box binding protein	NM_013684	5'-TGCCCAGCATCACTATTTCA-3'	5'-CAGATCTTCCCTAGAGCATCCT-3'

Supplemental Table S2: Genes differentially expression in prefrontal cortex, cerebral cortex, amygdala and hippocampus with CMS

Gene symbol	Gene name	Refseq	GCRMA *p*-value	Fold Change
		Amygdala		
Astx	amplified spermatogenic transcripts X encoded 5	NR_152851.1	0.0029	16.60
Mettl11a	methyltransferase like 11A	NM_001356433.1	0.0041	13.22
Psg28	pregnancy-specific glycoprotein 28	NM_054063.4	0.0093	12.89
S100a8	small calcium-binding protein A8	NM_013650	0.0268	7.85
S100a9	small calcium binding protein A9	NM_009114	0.0111	7.01
Vwf	von Willebrand factor	NM_011708	0.0088	6.14
Ccrl1	chemokine (C-C motif) receptor-like 1	NM_145700	0.0102	5.52
Sppl3	signal peptide peptidase like 3	NM_029012	0.0126	4.36
Sdr39u1	short chain dehydrogenase/reductase family 39U, member 1	NM_001082975	0.0222	4.06
Ndfip2	Nedd4 family interacting protein 2	NM_029561	0.0088	3.94
Ttr	transthyretin	NM_013697	0.0135	0.05
Pmch	pro-melanin-concentrating hormone	NM_029971	0.0029	0.06
Vgll3	vestigial like 3	NM_028572	0.0029	0.08
Gh	growth hormone	NM_008117	0.0062	0.10
Trhr	thyrotropin-releasing hormone receptor	NM_013696	0.0084	0.11
C1ql2	complement component 1, q subcomponent-like 2	NM_207233	0.0055	0.14
Grp	gastrin-releasing peptide	NM_175012	0.0119	0.14
Lhx9	LIM homeobox 9	NM_001025565.2	0.0231	0.15
Prlr	prolactin receptor1	NM_011169.5	0.0119	0.16
Irf2bp2	interferon regulatory factor 2 binding protein 2	NM_001164598.2	0.0254	0.17

		Hippocampus		
Shox2	short stature homeobox 2	NM_013665	0.0106	17.22
Tcf7l2	transcription factor 7-like 2 (T-cell specific, HMG-box)	NM_001142918	0.0116	8.53
Ptbp1	polypyrimidine tract binding protein 1	NM_008956	0.0109	6.51
Inadl	inactivation no after-potential D-like protein	NM_172696	0.0086	6.39
Synpo2	synaptopodin 2	NM_001191963	0.0080	5.82
Zic1	Zic family member 1	NM_009573	0.0029	5.09
Tnnt1	troponin T type 1	NM_011618	0.0119	5.04
Baiap3	brain-specific angiogenesis inhibitor 1-associated protein 3	NM_001163270	0.0304	4.88
Mettl11a	methyltransferase like 11A	NM_170592	0.0107	4.36
Sema3f	semaphorin-3F	NM_011349	0.0086	4.20
Dusp28	dual specificity phosphatase 28	NM_175118	0.0143	0.26
Fmod	fibromodulin	NM_021355	0.0119	0.26
Npas4	neuronal PAS domain protein 4	NM_153553	0.0123	0.29
Dync1li1	dynein, cytoplasmic 1, light intermediate chain 1	NM_146229	0.0107	0.32
Tshz2	teashirt zinc finger homeobox	NM_080455	0.0110	0.34
Egr2	early growth response 2	NM_010118	0.0110	0.36
Cyp20a1	cytochrome P450, family 20, subfamily A, polypeptide 1	NM_030013	0.0107	0.37
Ier5l	immediate early response 5-like	NM_030244	0.0392	0.38
Fos	FBJ murine osteosarcoma viral oncogene homolog	NM_010234	0.0103	0.39
Phkg1	phosphorylase kinase, gamma 1	NM_011079	0.0150	0.40
		Prefrontal Cortex		
Ngp	neutrophilic granule protein	NM_008694	0.0073	39.30

Ltf	lactotransferrin	NM_008522	0.0136	19.21
Camp	cathelicidin antimicrobial peptide	NM_009921	0.0198	16.20
S100a8	small calcium-binding protein A8	NM_013650	0.0073	11.86
Lcn2	Lipocalin-2, Neutrophil gelatinase-associated lipocalin precursor	NM_008491	0.0126	11.67
S100a9	small calcium binding protein A9	NM_009114	0.0034	10.46
Chi3l3	chitinase 3-like 3	NM_009892	0.0453	8.60
Cbr2	Carbonyl reductase 2	NM_007672	0.0167	8.54
Plunc	Palate, lung, and nasal epithelium carcinoma associated protein	NM_011126	0.0341	8.41
Acbd7	acyl-Coenzyme A binding domain containing	NM_030063	0.0407	6.57
Ttr	transthyretin	NM_013697	0.0001	0.01
Irs4	insulin receptor substrate 4	NM_010572	0.0277	0.12
Atf3	activating transcription factor 3	NM_007498	0.0332	0.23
Fosb	FBJ murine osteosarcoma viral oncogene homolog B	NM_008036	0.0115	0.24
Kl	klotho	NM_013823	0.0279	0.25
Cyr61	IGFBP-10,cysteine-rich, angiogenic inducer, 61	NM_010516	0.0329	0.33
Clspn	claspin homolog	NM_175554	0.0473	0.36
Fos	FBJ murine osteosarcoma viral oncogene homolog	NM_010234	0.0037	0.42
Baiap3	BAI1-associated protein	NM_001163270	0.0364	0.42
Rab14	RAB14, member RAS oncogene family	NM_016322	0.0407	0.43
		Cerebral Cortex		
S100a9	small calcium binding protein A9	NM_009114	0.0040	13.89
S100a8	small calcium-binding protein A8	NM_013650	0.0158	12.10

Drd2	dopamine receptor D21	NM_010077	0.0358	8.10
Tmem90a	transmembrane protein 91	NM_001033334	0.0040	6.64
Pde10a	phosphodiesterase 10A	NM_011866	0.0218	6.59
Brd4	bromodomain containing 41	NM_020508	0.0087	6.52
Rxrg	retinoid X receptor, gamma	NM_009107	0.0259	6.32
Pex5l	peroxisomal biogenesis factor 5	NM_021483	0.0491	6.17
Fam102b	family with sequence similarity 102, member B	NM_001163568	0.0377	5.77
Tmem90a	transmembrane protein 90A	NM_001105579	0.0087	5.35
Ttr	transthyretin	NM_013697	0.0037	0.01
Kl	klotho	NM_013823	0.0036	0.04
Kcne2	potassium voltage-gated channel, Isk-related family, member 2	NM_134110	0.0035	0.05
Clic6	chloride intracellular channel 6	NM_172469	0.0088	0.05
Folr1	folate receptor 1 (adult)	NM_008034	0.0118	0.06
Sostdc1	sclerostin domain containing 1	NM_025312	0.0099	0.08
Calml4	calmodulin-like 4	NM_138304	0.0118	0.09
Prlr	prolactin receptor1	NM_011169	0.0291	0.10
Kcnj13	potassium inwardly-rectifying channel, subfamily J, member 13	NM_053608	0.0130	0.12
Cldn2	claudin 2	NM_016675	0.0195	0.14

Supplemental Table S3: Combined Four Brain Parts Gene Set Enrichment Analysis (GSEA) for CMS-treated and Control Mouse Enriched Hallmarks

	Amy			Hippo			CC			PFC		
	NES	Normalized p-value	FDR q-value	NES	Normalized p-value	FDR q-value	NES	Normalized p-value	FDR q-value	NES	Normalized p-value	FDR q-value
INTERFERON_ALPHA_RESPONSE	1.1949844	0.18181819	0.5485827	NA	NA	NA	1.5238925	0.02053388	0.04102738	1.4057144	0.03599374	0.13118502
INTERFERON_GAMMA_RESPONSE	1.3393779	0.0238342	0.2241013	NA	NA	NA	1.0793613	0.29744527	0.39951953	1.3024528	0.05221519	0.15935773
HEDGEHOG_SIGNALING	NA	NA	NA	1.5523996	0	0.00669643	NA	NA	NA	NA	NA	NA
E2F_TARGETS	1.1560118	0.1745531	0.4932109	NA	NA	NA	1.4891485	0.0019084	0.04556593	1.2055364	0.12426036	0.26067472
UV_RESPONSE	1.1771957	0.1782284	0.5035012	NA	NA	NA	1.7186596	0	0.00935707	NA	NA	NA
OXIDATIVE_PHOSPHORYLATION	NA	NA	NA	NA	NA	NA	2.4545264	0	0	2.3004322	0	0
G2M_CHECKPOINT	0.926658	0.65726227	0.872483	NA	NA	NA	1.4702443	0.00770713	0.05037841	0.8722858	0.7714702	0.77198696
MITOTIC_SPINDLE	1.342231	0.01549587	0.27221018	NA	NA	NA	0.9535971	0.5555556	0.6462578	NA	NA	NA
HEME_METABOLISM	0.75689393	0.93157893	0.9761383	NA	NA	NA	1.3694173	0.03183521	0.0914392	1.0102863	0.41343284	0.57255167
P53_PATHWAY	-1.074966	0.20930232	0.46706864	-0.2227299	-0.6545895	0.998	1.0605545	0.3216912	0.42819828	-1.8520246	0	0.00191985
TNFA_SIGNALING/NFKB	0.7099219	0.9728318	0.98634404	-0.4344704	-1.2754439	0.002	-1.7944107	0	0.00452296	-2.0132668	0	0.00125

Amy, amygdala; Hippo, hippocampus; CC, cerebral cortex; PFC, prefrontal cortex; NES, normalized enrichment score; FDR, false discovery rate

NA, not available due to without enrichments.

Supplemental Table S4: Gene Enrichment Analysis for CMS vs. Control Mouse Model in Amygdala, Hippocampus, Cerebral Cortex and Prefrontal Cortex

Enrichment	Amy		Hippo		CC		PFC	
	p-value	FDR	p-value	FDR	p-value	FDR	p-value	FDR
Process networks								
Signal transduction_Cholecystokinin signaling	7.165E-08	1.060E-05	6.187E-01	7.617E-01	9.872E-02	3.067E-01	2.921E-01	5.360E-01
Reproduction_Feeding and neurohormone signaling	9.759E-05	3.611E-03	1.107E-02	2.569E-01	7.904E-03	7.542E-02	4.982E-01	5.360E-01
Immune response_Th17-derived cytokines	2.472E-01	5.462E-01	1.141E-02	2.569E-01	2.035E-04	2.971E-02	2.443E-04	1.759E-02
DNA damage_Checkpoint	1.014E-01	3.217E-01	3.068E-01	6.749E-01	1.498E-01	3.706E-01	6.002E-04	2.161E-02
Inflammation_Amphoterin signaling	9.040E-04	1.672E-02	2.123E-02	2.569E-01	7.187E-04	4.372E-02	5.493E-02	4.944E-01
Cell adhesion_Integrin-mediated cell-matrix adhesion	1.235E-03	2.031E-02	3.021E-01	6.749E-01	2.812E-01	5.002E-01	5.049E-01	5.360E-01
Transcription_Nuclear receptor transcriptional regulation	6.534E-02	2.480E-01	2.554E-01	6.168E-01	1.407E-03	4.372E-02	4.725E-01	5.360E-01
Reproduction_Gonadotropin regulation	3.312E-02	1.801E-01	5.381E-01	7.586E-01	1.639E-03	4.372E-02	3.453E-03	8.286E-02
Development_Blood vessel morphogenesis	2.158E-03	2.904E-02	1.465E-01	5.543E-01	1.743E-01	3.766E-01	5.526E-03	9.946E-02
Signal transduction_WNT signaling	9.467E-01	9.867E-01	2.124E-01	6.168E-01	2.585E-03	4.372E-02	4.400E-01	5.360E-01
Disease								
Central Nervous System Diseases	1.543E-23	2.859E-20	3.669E-05	5.775E-04	4.507E-17	5.312E-15	5.933E-06	6.526E-05
Brain Diseases	3.087E-23	2.860E-20	1.164E-05	2.932E-04	2.057E-16	1.996E-14	2.033E-06	2.702E-05
Mental Disorders	1.103E-22	6.811E-20	1.055E-04	1.264E-03	1.674E-17	2.302E-15	1.921E-07	5.220E-06
Psychiatry and Psychology	1.487E-22	6.890E-20	3.354E-05	5.775E-04	4.157E-17	5.277E-15	2.388E-07	5.807E-06
Rectal Disease	4.938E-21	1.525E-18	5.526E-08	3.479E-05	2.089E-21	2.038E-18	6.185E-06	6.559E-05

Congenital, Hereditary, and Neonatal Disease and Abnormalities	2.451E-21	9.084E-19	8.636E-07	8.505E-05	1.500E-13	6.543E-12	1.356E-05	1.171E-04
Gastrointestinal Neoplasms	9.597E-20	1.778E-17	1.587E-06	1.176E-04	2.470E-21	2.038E-18	1.405E-05	1.198E-04
Colorectal Neoplasms	1.076E-19	1.812E-17	1.705E-07	4.581E-05	5.737E-21	2.756E-18	4.581E-07	9.094E-06
Inteastinal Neoplasms	1.298E-19	2.004E-17	1.819E-07	4.581E-05	6.680E-21	2.756E-18	4.724E-07	9.094E-06
Heredodegenerative Disorder, Nervous System	2.476E-20	6.553E-18	1.090E-03	7.929E-03	4.843E-19	1.169E-16	1.879E-02	3.911E-02
GO Processes								
Nervous System Development	5.041E-34	3.511E-30	1.844E-11	7.278E-08	3.732E-24	2.069E-20	2.481E-02	7.965E-02
System Development	2.383E-30	8.300E-27	1.924E-08	7.593E-06	7.012E-18	3.529E-15	1.937E-02	6.886E-02
Neurogenesis	3.080E-28	7.150E-25	5.333E-08	1.403E-05	1.436E-19	1.496E-16	4.114E-02	1.045E-01
Generation of Neurons	4.503E-27	7.840E-24	3.932E-07	5.697E-05	1.672E-17	5.794E-15	1.507E-01	2.183E-01
Response to Organic Substance	7.283E-27	1.015E-23	8.616E-05	1.790E-03	2.512E-15	4.686E-13	3.538E-06	1.086E-04
Multicellular Organism Development	9.583E-27	1.112E-23	2.006E-07	3.959E-05	6.036E-16	1.455E-13	3.293E-02	9.319E-02
Neuron Differentiation	1.855E-24	1.846E-21	1.533E-03	1.111E-02	5.397E-12	3.563E-10	1.658E-01	2.321E-01
Central Nervous System Development	2.463E-24	2.145E-21	1.062E-08	5.988E-06	9.048E-20	1.254E-16	3.096E-02	8.957E-02
Anatomical Structure Development	3.085E-24	2.267E-21	3.707E-08	1.126E-05	2.001E-13	2.158E-11	3.336E-02	9.383E-02
Regulation of Biological Quality	3.255E-24	2.267E-21	1.029E-05	3.984E-04	8.901E-15	1.299E-12	3.757E-03	2.441E-02

Supplemental Table S5: Characteristics of patients with MDD and control individuals in the Ttr testing cohort.

Sample ID	Sex	Age	BDI	Diagnosis
1	Female	27		MDD
2	Female	33		MDD
3	Female	44		MDD
4	Female	44		MDD
5	Female	45		MDD
6	Female	53		MDD
7	Female	54		MDD
8	Female	54		MDD
9	Female	61		MDD
10	Male	22		MDD
11	Male	48		MDD
12	Male	52		MDD
13	Female	30	0	NA
14	Female	31	0	NA
15	Female	31	3	NA
16	Female	38	0	NA
17	Female	39	1	NA
18	Female	45	4	NA
19	Female	55	0	NA
20	Female	60	4	NA

21	Female	61	2	NA
22	Male	23	7	NA
23	Male	38	11	NA
24	Male	59	1	NA

BDI, Beck Depression Inventory

Supplemental Figures:

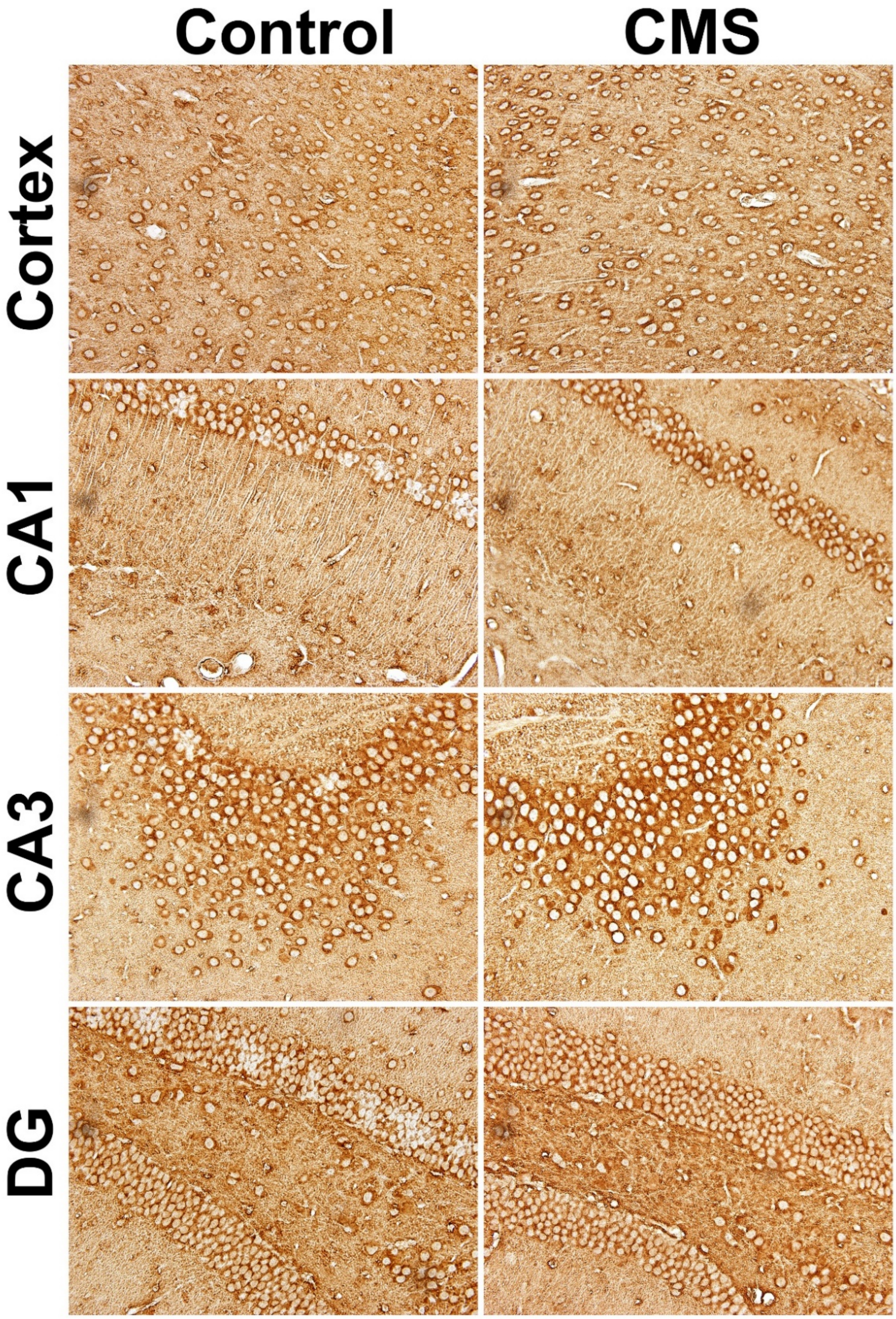

Supplemental Figure S1: Immunohistochemical staining of Tau-1 for cerebral cortex and hippocampal CA1, CA3, and dentate gyrus (DG) in chronic mild stress (CMS) treated and control mice. Scale bar, 50 μ m.

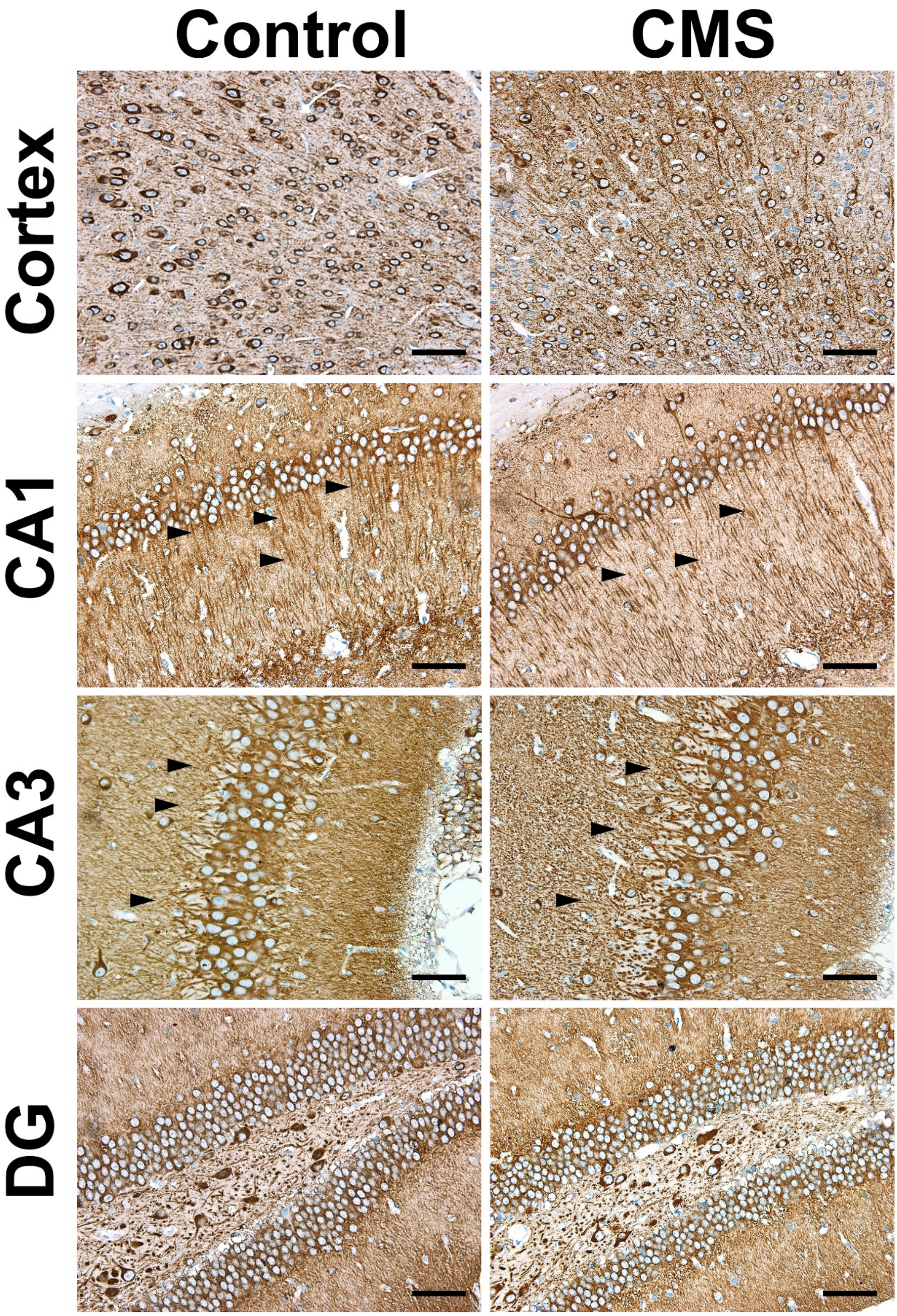

Supplemental Figure S2: Immunohistochemical staining of MAP2 for cerebral cortex and hippocampal CA1, CA3, and dentate gyrus (DG) in chronic mild stress (CMS) treated and control mice. Arrow heads represent dendrites. Scale bar, 50 μm.
